# Supplementary material for: Stretch-activated ion channels identified in the touch-sensitive structures of carnivorous Droseraceae plants
Source: eLife. 2021 Mar 16;10:e64250. doi: 10.7554/eLife.64250 (PMC7963481; doi:10.7554/eLife.64250)
Supplement: Figure 1—source data 1. [file elife-64250-fig1-data1.docx]

**Figure 1- source data 1. Size estimates of two *Arabidopsis* (Col-0) samples compared to our Venus flytrap strain (CP01).**

| Species | DNA Content (pg/2C) | St. Dev. |
| --- | --- | --- |
| ***A. thaliana* (Col-0)**  **Sample #1** | 0.38 | 0.010 |
| ***A. thaliana* (Col-0)**  **Sample #2** | 0.40 | 0.007 |
| **D. muscipula (CP01)** | 7.86 | 0.359 |
