# Supplementary material for: Stretch-activated ion channels identified in the touch-sensitive structures of carnivorous Droseraceae plants
Source: eLife. 2021 Mar 16;10:e64250. doi: 10.7554/eLife.64250 (PMC7963481; doi:10.7554/eLife.64250)
Supplement: Figure 1—source data 2. [file elife-64250-fig1-data2.docx]

| Figure 1- source data 2. Summary of sequencing reads used to build the *de novo* transcriptome (NCBI Transcriptome Shotgun Assembly Sequence Database accession # GHJF00000000). | | | | |
| --- | --- | --- | --- | --- |
| Sample | Paired replicates | Tissue | # paired reads | SRA accession # |
| unfed1 | no | 2 traps | 9,079,584 | SRR8834216 |
| unfed2 | no | 2 traps | 9,424,911 | SRR8834215 |
| fed1 | no | 2 traps | 9,597,638 | SRR8834218 |
| fed2 | no | 2 traps | 9,887,029 | SRR8834217 |
| trap1 | yes (A) | ~20 traps^a^ | 4,410,249 | SRR8834220 |
| trigger_hair1 | yes (A) | 250 hairs | 3,702,412 | SRR8834221 |
| trap2 | yes (B) | ~20 traps^a^ | 10,974,314 | SRR8834219 |
| trigger_hair2 | yes (B) | 750 hairs | 9,711,759 | SRR8834214 |
| trap3 | yes (C) | ~20 traps^a^ | 11,418,472 | SRR8834222 |
| trigger_hair3 | yes (C) | 750 hairs | 9,975,164 | SRR8834213 |
| ^a^Trigger hairs removed. | | | | |
