# Supplementary material for: Stretch-activated ion channels identified in the touch-sensitive structures of carnivorous Droseraceae plants
Source: eLife. 2021 Mar 16;10:e64250. doi: 10.7554/eLife.64250 (PMC7963481; doi:10.7554/eLife.64250)
Supplement: Transparent reporting form [file elife-64250-transrepform.docx]

***eLife’s* transparent reporting form**

We encourage authors to provide detailed information *within their submission* to facilitate the interpretation and replication of experiments. Authors can upload supporting documentation to indicate the use of appropriate reporting guidelines for health-related research (see [EQUATOR Network](http://www.equator-network.org/%20)), life science research (see the [BioSharing Information Resource](https://biosharing.org/)), or the [ARRIVE guidelines](http://www.plosbiology.org/article/info:doi/10.1371/journal.pbio.1000412) for reporting work involving animal research. Where applicable, authors should refer to any relevant reporting standards documents in this form.

If you have any questions, please consult our Journal Policies and/or contact us: [editorial@elifesciences.org](mailto:editorial@elifesciences.org).

**Sample-size estimation**

- You should state whether an appropriate sample size was computed when the study was being designed
- You should state the statistical method of sample size computation and any required assumptions
- If no explicit power analysis was used, you should describe how you decided what sample (replicate) size (number) to use

Please outline where this information can be found within the submission (e.g., sections or figure legends), or explain why this information doesn’t apply to your submission:

**Gene expression analysis:** Differential gene expression analyses by RNA-sequencing or qRT-PCR were performed on samples collected in triplicate or more. This is typical in the field for such experiments. Our de novo transcriptome build was generated using additional treatment samples collected in duplicate to increase the number of transcripts detected. All replicate information is provided in the results, materials and methods, and supplementary material.

**Electrophysiology:** Sample size in our study indicates number of cells tested for all electrophysiological recording. Ns were determined based on previous publications from the lab and experience. A minimum of N=4 was the lowest number of cells included for each data set. Number of cells tested for each condition is mentioned in figure legends and in figure supplement data.

**Replicates**

- You should report how often each experiment was performed
- You should include a definition of biological versus technical replication
- The data obtained should be provided and sufficient information should be provided to indicate the number of independent biological and/or technical replicates
- If you encountered any outliers, you should describe how these were handled
- Criteria for exclusion/inclusion of data should be clearly stated
- High-throughput sequence data should be uploaded before submission, with a private link for reviewers provided (these are available from both GEO and ArrayExpress)

Please outline where this information can be found within the submission (e.g., sections or figure legends), or explain why this information doesn’t apply to your submission:

**Gene expression analysis:** Samples for RNA-sequencing were collected over multiple days. To account for possible batch effects, paired biological replicates were collected. Here, “biological replicates” infers that sample replicates were generated from different plants or plant leaf tissue. Raw RNA-sequencing read files, as well as differential gene expression analysis files and our de novo transcriptome build, are publicly available through the relevant NCBI databases. Information is provided in the materials and methods.

**Electrophysiology:** Each dataset is a compilation of at least two independent transfections, done on different days, for all electrophysiological recordings. The only time a cell was excluded was if the quality of the recording was poor and the leak current was very high. Necessary information is included in the materials and methods section.

**Statistical reporting**

- Statistical analysis methods should be described and justified
- Raw data should be presented in figures whenever informative to do so (typically when N per group is less than 10)
- For each experiment, you should identify the statistical tests used, exact values of N, definitions of center, methods of multiple test correction, and dispersion and precision measures (e.g., mean, median, SD, SEM, confidence intervals; and, for the major substantive results, a measure of effect size (e.g., Pearson's r, Cohen's d)
- Report exact p-values wherever possible alongside the summary statistics and 95% confidence intervals. These should be reported for all key questions and not only when the p-value is less than 0.05.

Please outline where this information can be found within the submission (e.g., sections or figure legends), or explain why this information doesn’t apply to your submission:

**Gene expression analysis:** Statistical analysis methods standard in the field are used for gene expression analyses. Methods and statistical test results are presented in tables, figure legends, and described in the materials and methods.

**Electrophysiology:** Electrophysiology data presented in this manuscript is descriptive and reports channel properties. Since these properties have been described only for one gene, FLYC1, and has not been compared against either different genes or conditions, statistics have not been reported.

(For large datasets, or papers with a very large number of statistical tests, you may upload a single table file with tests, Ns, etc., with reference to sections in the manuscript.)

**Group allocation**

- Indicate how samples were allocated into experimental groups (in the case of clinical studies, please specify allocation to treatment method); if randomization was used, please also state if restricted randomization was applied
- Indicate if masking was used during group allocation, data collection and/or data analysis

Please outline where this information can be found within the submission (e.g., sections or figure legends), or explain why this information doesn’t apply to your submission:

**Gene expression analysis:** Plants sampled for gene expression analysis were grown under the specified greenhouse conditions. From these, random plants were sampled. See materials and methods.

**Electrophysiology:** For electrophysiology data, samples were allocated into different groups based on the different genes tested for the study, and reported against a negative control (mock transfected cells) and a positive control (PIEZO1).

**Additional data files (“source data”)**

- We encourage you to upload relevant additional data files, such as numerical data that are represented as a graph in a figure, or as a summary table
- Where provided, these should be in the most useful format, and they can be uploaded as “Source data” files linked to a main figure or table
- Include model definition files including the full list of parameters used
- Include code used for data analysis (e.g., R, MatLab)
- Avoid stating that data files are “available upon request”

Please indicate the figures or tables for which source data files have been provided:

Figure 1- source data 1, Figure 1- source data 2
